# Association between sugar cane burning and acute respiratory illness on the island of Maui

**DOI:** 10.1186/s12940-015-0067-y

**Published:** 2015-10-07

**Authors:** Christina Louise Mnatzaganian, Karen L. Pellegrin, Jill Miyamura, Diana Valencia, Lorrin Pang

**Affiliations:** Skaggs School of Pharmacy and Pharmaceutical Sciences, University of California San Diego, 9500 Gilman Drive, MC 0764, La Jolla, CA 92093-0764 USA; The Daniel K Inouye College of Pharmacy, University of Hawaii at Hilo, 34 Rainbow Drive, Hilo, HI 96720 USA; Hawaii Health Information Corporation, 733 Bishop, Makai Tower, Ste 1870, Honolulu, HI 96813 USA; University of Hawaii-Manoa, Honolulu, HI 96826 USA; State of Hawaii Department of Health (Maui District Health Office), 54 High Street, Wailuku, HI 96793 USA

**Keywords:** Acute respiratory disease, Epidemiology, Smoke adverse effects, Sugar cane, Volcanic smog, Vog, Agricultural burning, Environmental

## Abstract

**Background:**

Sugar cane harvesting by burning on Maui island is an environmental health issue due to respiratory effects of smoke. Volcanic smog (“vog”) from an active volcano on a neighboring island periodically blankets Maui and could confound a study of cane smoke’s effects since cane burning is not allowed on vog days. This study examines the association between cane burning and emergency department (ED) visits, hospital admissions, and prescription fills for acute respiratory illnesses.

**Methods:**

This retrospective study controlled for confounders that could increase respiratory distress on non-burn days by matching each burn day with a non-burn day and then comparing the ratio of patients with respiratory distress residing in the path of sugar burn smoke to those residing elsewhere on Maui on burn versus non-burn days. Patients with acute respiratory distress were defined as those with one or more acute respiratory diagnoses at one of the hospitals or emergency departments on Maui. Separately, patients with acute respiratory illness were identified through prescription records from four community pharmacies, specifically defined as those who filled prescriptions for acute respiratory distress.

**Results:**

There were 1,256 reports of respiratory distress prescriptions and 686 hospital/ED diagnoses of acute respiratory illness. The ratio of cases within to outside of smoke exposure was higher on burn days for both the ED/hospital data and the pharmacy, though not statistically significant. In post-hoc analyses of the pharmacy data based on the number of acres burned as a proxy for volume of smoke, there was a dose response trend for acreage burned such that the highest quartile showed a statistically significant higher proportion of acute respiratory distress in the exposed versus non-exposed regions (*P* = 0.015, OR 2.4, 95 % CI [1.2–4.8]).

**Conclusions:**

After adjusting for confounders on non-burn days, there was a significantly higher incidence of respiratory distress in smoke-exposed regions when greater amounts of acres were burned. Health officials should consider actions to reduce the negative health outcomes associated with sugar cane burning practices.

## Background

Outdoor air pollution is associated with mortality and hospital admissions due to respiratory and cardiovascular disease in both short-term and long-term studies [1]. There is a paucity of research on the specific impact of sugar cane burning on health, despite evidence that it is at least as toxic as pollution produced by traffic and more toxic than traffic after repeated exposures [2, 3]. In particular, no published studies have examined the impact of current sugar cane burning practices on the island of Maui in Hawaii.

Sugar plantations were established two centuries ago on the islands of Hawaii. Today, only the island of Maui continues to produce 200,000 tons of cane annually [4]. Controlled, scheduled burns of cane fields occur prior to harvest to reduce the volume of waste material for transport and processing. Cane field fires produce smoke and ash above the Maui central valley resulting in ash fall locally known as “Maui snow”. Burns typically begin in the early morning hours and end before dawn, thereby avoiding peak traffic periods and school or church sessions.

Burning rules are set and monitored by the Hawaii Department of Health (DOH). Burning is prohibited on days when high amounts of volcanic smog (“vog”) occur on Maui, originating from the consistently-active Kilauea Volcano on the Big Island of Hawaii, located 117 miles southeast of Maui. There is a general northeasterly wind direction during days when cane burning is allowed. When vog blows in on weaker southerly winds, it tends to uniformly expose Maui’s entire island population. Therefore, vog could confound clinical/epidemiologic studies of cane burning’s respiratory effects if one used days of no cane burning as controls for days of burning. Additionally, cane cannot be burned during extremely rainy weather, which is associated with cold symptoms, another potential confounder. Therefore, comparisons of burn versus non-burn days on Maui require methods that control for at least these confounders.

The study of sugar cane burning on Maui is a particularly important environmental health issue given the relatively high prevalence of asthma in Hawaii. According to the National Center for Environmental Health, asthma affects 16.9 % of children and 16.1 % of adults in Hawaii, higher than the US prevalence of 12.5 % and 13.3 %, respectively [5, 6]. Asthma exacerbations have continually contributed to increasing emergency department (ED) visits, hospitalizations, and mortality [7]. Hawaii reports approximately 5,000 ED visits and 1,500 hospital admissions annually due to asthma exacerbations [8]. According to the National Institute of Health, environmental factors that may trigger or exacerbate acute respiratory illnesses include viral respiratory infections, environmental allergens, smoking, exercise, occupational chemicals, environmental changes, irritants, emotions, stress, drugs, food, changes in weather, exposure to cold air, and endocrine and comorbid conditions [9]. Cane burning and vog emissions are typical examples of smoke, chemical, and particulates on Maui. Additionally, residents may be exposed to molds and viral infections during extremely wet and rainy weather when cane burning is not permitted.

There are only a few published studies to date that examine the health effects of sugar cane burning and none examining health effects under current DOH burning restrictions in the unique environment of Hawaii. Lehman studied 36 patients who were scheduled for intracutaneous skin testing due to significant chronic allergy problems. When exposed to sugar cane smoke extract, there was a significant increase (*P* < 0.01) in positive skin reactions in subjects exposed to cane smoke extract as compared to the control group [10]. In Louisiana, there were 6,498 hospital visitations for asthma during a two-year study period with a positive (though not significant) dose–response trend in asthma hospitalization rates during sugar cane burning [11].

Brazilian studies demonstrate an association with adverse health effects. One study analyzed 673 records of children less than thirteen years old and elderly greater than 64 years old and found particulate matter (PM) and carbon monoxide (CO) markers from cane burning led to respiratory effects [12]. Data analysis showed that the PM_10_ during the nonburning period was 28.9 ± 12.8 μg/m^3^ compared to 87.7 ± 57.9 μg/m^3^ during the burning period. The PM_2.5_ during the nonburning period was 10.0 ± 4.6 μg/m^3^ compared to 22.8 ± 14.7 μg/m^3^ during the burning period. In both the child and elderly group, PM_10_, PM_2.5_, and black carbon were significantly associated with respiratory hospital admissions even after adjusting for season and weather. These correlations remained significant even after adjusting for season and weather.

In another ecological time-series study, total suspended particle (TSP) concentrations from cane burning doubled during burn periods with a statistically significant increase in asthma hospital admissions 1–5 days after increasing TSP concentrations [13]. Study authors used a lag structure of 0 to 9 days between burn dates and hospital admissions with adjustments for over-dispersion. During the 493 days of the study, there were a total of 640 asthma hospital admissions; during the burning period (318 days) there were 477 admissions, a rate 50 % higher, and statistically significant, compared to 163 admissions during the non-burning period (175 days).

In a descriptive, cross-sectional study of 1,076 private- and public-schooled children aged 10–14, Riguera found lower asthma symptoms but higher rates of rhinitis in children during times of burning [14]. Through a series of assessments, he found that the prevalence of asthma and rhinitis symptoms was 11 % and 33.2 %, respectively. Rhinitis occurred most frequently from June to October, a period that matches the sugar cane harvest season in Brazil as well as seasonal variations. Additionally, daily prevalence of peak expiratory flow below 20 % of the median of each child’s best measurements was greater in days with higher PM_2.5_ concentrations. The authors concluded that the prevalence of asthma symptoms was actually below the national Brazilian average (19 %) whereas rhinitis prevalence exceeded the national average (29.6 %) during the study period.

More recently, there is evidence that reduction of pre-harvest sugar cane burning in response to a state law in Brazil requiring the gradual elimination of this practice has been associated with a decrease in hospitalizations due to respiratory disease [15].

In 1972, the United States Environmental Protection Agency (EPA) studied cane and leaf burning from Hawaii crops in an incinerating tower for pollutant analysis [16]. Burning of 20 whole cane plots and 19 cane leaf trash samples yielded particulates, carbon monoxide, and hydrocarbon emissions all within normal ranges of many other herbaceous types of fuel previously burned within the tower. During whole cane burns, particulate yields averaged 112 lbs/acre of fuel burned (99 % CI with true mean between 92 and 132 lbs/acre), which was determined to not be excessive. The carbon monoxide yield averaged 1,113 lbs/acre (99 % CI with true mean between 843 and 1,383 lbs/acre), and also determined to be a moderate amount emitted, similar to the yield from dry cereal grain straw. Finally, the hydrocarbon yield averaged 152 lbs/acre (99 % CI with true mean of 121 lbs/acre) and authors concluded this also to be within normal emissions. Pollutant yields from leaf trash were a little less than from whole cane; additionally, those fires simulated against the direction of wind flow did not emit any significant difference compared those in the direction of wind flow. However, this marker study does not address all pollutants potentially causing health problems or examine if mixtures of pollutants have a compounding effect. For example, vog is another source of air pollution on Maui and was studied at a clinic downwind from the active volcano on the Big Island of Hawaii [17]. In sampling 1,189 patients, high vog exposure during increased volcanic activity was associated with a six-fold increase in health visits for acute airway problems, cough, headache, and pharyngitis.

Given the link between sugar cane burning and negative respiratory health outcomes in other locations, the high prevalence of asthma in Hawaii, and the complex environment in Hawaii, it is important to study the impact of current sugar burning practices on Maui. Therefore, this study retrospectively examines the relationship between sugar cane burning and acute respiratory illnesses on the island of Maui on burn days while controlling for confounding factors likely to be present on non-burn days.

## Methods

### Study design

This study used a historical, controlled (paired), and partially blinded design to compare the ratio of respiratory distress events among those residing in the path of sugar cane burn smoke to those residing elsewhere on Maui on burn versus non-burn days. This design controls for confounding variables (e.g., vog and rain) on the non-burn days. The ratio of cases from smoke exposure area to non-exposed areas was hypothesized to be higher on burn days compared to non-burn days. The Institutional Review Boards of the University of Hawaii and the Hawaii DOH granted ethical approval.

### Data sources

Cane burning records between April 2011-April 2012 were obtained from the DOH. This period was selected because cane employees used a newer, detailed system of reporting information with spot-check confirmation by DOH personnel. Investigators selected 55 burn days where burning was completed by 6:00 AM (to ensure residential exposures only), more than 70 acres were burned, and uniform wind speed greater than zero. Each burn day was matched with a random non-burn day that was preceded by at least four days of no burning as a “washout” period.

DOH field maps and Google Earth software were then utilized to identify areas where burning occurred. The sugar cane company maps and numbers all cane fields so the burn location and acreage is available in records submitted to the DOH. Investigators plotted the site of the burn and drew a cone of smoke exposure based on the averaged wind directions with a 30 degree angle of smoke dispersion. This angle was determined from observations of smoke from the sugar mill during a variety of wind conditions as well as previously conducted research [18]. Areas of exposure were drawn to the edge of the island.

Respiratory distress events on selected burn and non-burn dates were obtained from two sources: a database of all hospital admissions and all ED visits on Maui and four community pharmacies on Maui. Hospital admission and ED data pertaining to acute respiratory disease were obtained from Hawaii Health Information Corporation (HHIC). HHIC collects, cleans, and verifies detailed patient-level discharge data from hospitals for all payers in the state of Hawaii. HHIC data elements include patient race/ethnicity, age, gender, insurer, length of stay, patient residence, and primary and secondary International Classification of Diseases, 9^th^ revision (ICD-9) diagnostic codes. Specifically, the following discharges and ED visits were obtained from HHIC on selected burn and non-burn days: asthma with exacerbation (ICD-9 493.02), acute bronchitis (ICD-9 466.0), acute sinusitis (ICD-9 461), acute pharyngitis including streptococcal pharyngitis (ICD-9 462; 034.0), acute conjunctivitis (ICD-9 372.0), cough (ICD-9 786.2), headache (ICD-9 339), all pneumonias (ICD-9 480–486), and other acute airway problems (requiring immediate treatment such as oxygen, medications, or respiratory treatments, ICD-9 786.05).

Databases of prescriptions filled from four community pharmacies in four different regions (representing 13 % of the pharmacies on Maui) were queried for the following medications used to treat respiratory conditions: albuterol, ipratropium, and albuterol/ipratropium inhalers and nebulizers, nasal sprays including fluticasone, mometasone, and flunisolide, promethazine/codeine syrup, benzonatate caplets (100 mg and 200 mg), prednisone tablets (5 mg, 10 mg, and 20 mg), methylprednisolone dose-packs, and medications for conjunctivitis including prednisolone, olopatadine, and prednisolone eye drops.

For each respiratory distress event (i.e., hospital admission, ED visit, prescription filled), the patient’s home address was obtained. Patients with residences outside of Maui were excluded, and those with multiple qualifying diagnoses or prescriptions were counted as one exposure.

**Fig. 1:**
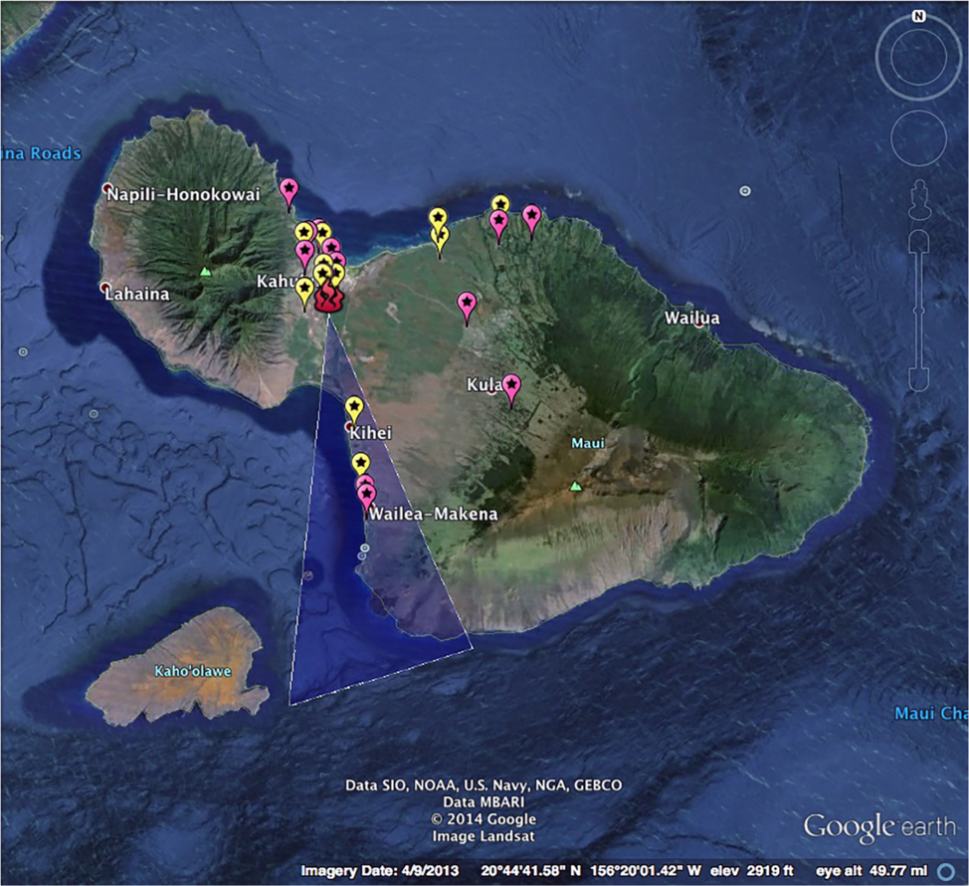
Example exposures on Maui map of subjects presenting on given burn and matched non-burn day. Legend: Red fire icon = sugar cane burn site; Blue triangle = 30 degree angle of smoke exposure; Yellow = subjects presenting on given burn day; Pink = subjects presenting on matched non-burn day

For each burn day and matched non-burn day, subjects with one or more respiratory distress events were plotted to reside either in the area of cane smoke exposure or outside of it. To facilitate understanding of the design, subjects from both burn days and non-burn days were plotted on the same map (Fig. 1). Data were tallied across all pairs of days retaining the exposed vs. non-exposed and burn day vs. non-burn day designations. A 2x2 Chi-square test was used to compare these mutually exclusive counts, aggregated across all burn/non-burn day pairs. An equivalent approach would be to take the odds ratio (OR) of these two ratios with a null hypothesis of no effect having this OR (burn day ratio: non-burn day ratio) = 1.

## Results

There were 191 burn days from April 2011-April 2012; 55 burn days and 55 matching non-burn days were chosen based on the criteria previously detailed (Table 1).

**Table 1:** Hospital/ED and pharmacy data by temporal exposure only

	Cases burn day	Cases non-burn day	RR (95 % CI) with base population of 10,000
Hospital/ED^a^	315	371	1.17 (1.01, 1.36)
Pharmacy^b^	576	680	1.18 (1.06, 1.31)

^a^ *p* = 0.04

^b^ *p* = 0.0025

### Hospital data

Island-wide, there were 315 hospital/ED acute respiratory visits on burn days and 371 hospital/ED visits on non-burn days (*N* = 686); without any mapping to smoke exposure versus no exposure there was a significantly higher (*P* = 0.04) rate of respiratory illnesses on the days when cane was not burned (Table 1). However, in the analyses of exposed vs. non-exposed smoke areas to control for the confounders on non-burn days, 37 (11.7 %) of the 315 cases on burn days were exposed compared to 34 (9.2 %) of the 371 cases from the corresponding areas during non-burn days (Table 2). This higher proportion of respiratory distress from exposed areas on burn days was not significant (*P* = 0.33; RR = 1.28, 95 % CI [0.82–2.0]).

**Table 2:** Hospital and pharmacy data by temporal and direction of wind exposure

	Cases within area of smoke exposure	Cases outside area of smoke exposure	Total cases	Ratio of cases within to outside of smoke exposure
Hospital/ED data				
Burn day	37 (11.7 %)	278 (88.3 %)	315	0.133
Non-burn day	34 (9.2 %)	337 (90.8 %)	371	0.101
*p* = 0.33; OR = 1.32 (95 % CI [0.81–2.16])			686	
*p*-value compared with matched controls				
Pharmacy data				
Burn day	76 (13.2 %)	500 (86.8 %)	576	0.152
Non-burn day	78 (11.5 %)	602 (88.5 %)	680	0.130
*p* = 0.40; OR = 1.17, (95 % CI [0.84, 1.64])			1,256	
*p*-value compared with matched controls				

### Pharmacy data

Similar results were found with the pharmacy data. Island-wide, there were 576 patients who filled respiratory prescriptions on burn days and 680 subjects who filled prescriptions on non-burn days (*N* = 1,256); without mapping to smoke exposure areas there was a significantly higher number during non-burn days (*P* = 0.0025; RR 1.18, 95 % CI [1.06–1.31]) (Table 1). However, in smoke exposure mapping analyses of exposed vs. non-exposed areas to control for confounders on non-burn days, 76 (13.2 %) of the 576 cases on burn days were exposed compared to 78 (11.5 %) of the 680 cases in the corresponding areas during the non-burn days (Table 2). This greater proportion of respiratory distress in the area of exposure on burn days was not statistically significant.

Thus, the hypothesis that the ratio of respiratory distress events among those inside to outside smoke exposure areas would be greater on burn day than on non-burn days was not supported with either hospital data or pharmacy data. To explore the possibility of dose response in post-hoc analyses, the selected burn days (and paired non-burn days) were broken down by quartiles according to the acreage burned; in essence, into four smaller studies using data from the community pharmacies. There was a statistically significant (*P* = 0.02; OR = 2.4 (95 % CI [1.2–4.8]) higher proportion of illness from the smoke exposed areas during the highest quartile of cane burning based on number of acres burned. The remaining quartiles did not show statistically significant differences although the OR trended downwards as the amount of cane burned decreased by acreage (1.07, 0.86, 0.74, respectively) (Table 3).

**Table 3:** Trend analysis by acres burned

	Cases within area of smoke exposure	Cases outside area of smoke exposure	OR (95 % CI)	*P*-value compared with matched controls
Quartile 1: 108–163 acres				
Burn day	20	111	2.4 (1.2, 4.8)	0.02
Non-burn day	15	196		
Quartile 2: 91.1–107 acres				
Burn day	19	114	1.07 (0.6, 2.1)	0.87
Non-burn day	24	156		
Quartile 3: 76–91 acres				
Burn day	32	133	0.86 (0.5, 1.5)	0.67
Non-burn day	31	111		
Quartile 4: 69.7–75 acres				
Burn day	6	141	0.74 (0.3, 2.2)	0.79
Non-burn day	8	139		

To estimate study power and sample size, with 177 total exposures from each of the burn and non-burn days one could have detected a shift in cases from 15 to 11 % of total cases in the exposed areas between the burn and non-burn days, respectively (alpha error of 5 % and beta of 20 %). With 750 in each group one had the same power to detect a smaller 15 % to 13 % shift. Statistical calculations were done using EPIINFO 7.0; if not specified otherwise, P-values were calculated by Fishers Exact tests.

## Discussion

The use of global positioning system (GPS) mapping allowed study investigators to compare the ratio of cases of cane-smoke exposed regions during burning to the ratio of cases from these same areas when no burning occurred to isolate the effect of burning. Comparison of these two ratios adjusted for vog, as well as other geographical or temporal confounding effects. Without these adjustments we showed there was significantly more symptoms on the days when cane was not burned, which would be expected due to respiratory effects of vog and the prohibition of burning cane on vog days. With these adjustments there was an increase, although statistically insignificant, in the proportion of cases from smoke-exposed areas during days of cane burning. When this same analysis was repeated by quartiles of acreage of cane burned as a “dose” effect, there was a trend showing higher proportions from the smoke-exposed areas when more cane was burned. The highest quartile showed a statistically significant higher proportion corresponding to 2.4 fold increase in the expected number of cases and a dose–response effect by quantity of acreage burned.

When studying the health impact of cane burning, the typical design would tally all acute symptoms associated with a burn event and compare to control periods with no cane smoke exposure. It is typically assumed that most or all of the acute symptoms caused by cane burning occur temporally near the burn event. When symptoms (or the measurable events reflecting the symptoms) might lag the burn event by a few days, designs must account for this potential lag to ensure that the control period isn’t “contaminated” with lagged effects from the burn event. In this study, control periods were chosen to account for this potential lag in symptoms by selecting non-burn days that were preceded by at least four days of no burning. Similarly, designs that limit measurement of acute symptoms to the day of the burn event risk missing lagged symptoms that are caused by the event. While this approach was used in this study and represents a limitation, measuring symptoms in the first 24 h after exposure was believed to be the best way to capture most of those caused by the burn event while minimizing the inclusion of symptoms due to other causes.

A unique challenge to designing research on the health impact of cane burning on Maui is that burning is intentionally avoided on “voggy” days. Thus, simply comparing acute symptoms across the Maui population on burn versus non-burn days does not isolate the impact of cane burning. That is, the higher vog pollution on non-burn days potentially masks the impact of cane burning. Therefore, the design must adjust for vog and other potential confounding factors. One approach would be to select the control, no-burn days, to have exactly the same vog effect as the burn days. This is problematic as the declaration of a “vog day” is very subject and imprecise, depending on observing haze and the amount and duration of the haze seen. For a given year, different agencies report varying percentages of vog days between 5–20 % for Maui. Additionally, associations with marker chemical pollutant(s) are not well validated. Therefore, the method used in this study (ratio of ratios) is more likely to effectively control for confounders that potentially affect health island-wide by comparing the ratio of symptoms in exposed versus non-exposed regions on burn versus non-burn days.

Furthermore, it may appear that the lower two quartiles of acreage burned have a “protective effect” where areas of smoke exposure have proportionately fewer cases when looking at the trend effect based on acreage. It is possible that there is an underrepresentation of outcomes in all quartiles. Perhaps the methods of measuring respiratory distress only captured more acute events, so that the impact of burning on indicators of milder respiratory distress (e.g., clinic visits, taking a sick day from work, and/or general malaise) were not measured. For example, when the smoke exposure is mild (lower quartiles), respiratory distress symptoms may be milder, and subjects take a “wait and see” approach hoping that the symptoms clear with the smoke. This could result in lower than normal ED visits, hospital admissions, or prescription fills that day. The following day, the symptoms either clear or result in case outcomes if they don’t resolve; thus a lag-effect may exist. Our method only detected symptoms on the day of the burning and would miss second-day delayed visits and/or more mild distress if they occurred.

While we controlled for the effect of vog and rain more likely to occur on non-burn days, our study design can address other confounders like “healthy survivor” effect where residents relocate to another part of the island to avoid the vog as well as disproportionate pharmacy coverage across a geographical area. The strength of the methodology is that the adjustments occur without actually knowing the degree of confounding or even identifying that a confounder exists. This is analogous to having an individual be his own control, which adjusts for known and unknown confounders, measured or not. For small communities requiring a rapid assessment of outdoor pollution sources, this method should be considered. This method requires that the targeted confounding effect occurs either uniformly geographically (vog, pollen, or mold) or persists over time (pharmacy coverage, healthy survivor effect, etc.). An alternative approach to adjusting for confounders may involve mathematical modeling (regressions, etc.). In order to do this, one must measure the confounding effect upon which to build the model. For effects like vog, this may be difficult since there are a variety of irritants, acting alone and in combinations, and clinical effects may persist beyond the actual detection period of the polluting chemicals in the air. However, our design of comparing morbidity of self-matched groups offers a simple way to adjust for confounders without actually knowing the markers or the magnitude of their effects. Perhaps a stratified analysis on the matched case–control pairs would have offered more precision but it is not clear how much more these statistical methods would have added [19].

Given the accumulation of data showing the negative health impact from air pollution in general and sugar cane burning specifically, public health officials should consider actions to minimize this impact. This study suggests that limiting the number of acres burned on a given day might significantly reduce acute respiratory distress among those residing in exposed areas. It is noteworthy that in Brazil, where most of the studies of sugar cane burning impact have been conducted, one state has passed a law requiring the gradual elimination of pre-harvest burning. Preliminary results indicate that this has resulted in improvements in respiratory health [15]. While economic interests are often of concern with such actions, economic evaluations should include all likely impacts, including cost of associated healthcare, productivity, and quality of life. On Maui, where tourism is the dominant economic engine, impact on visitors should also be considered.

## Conclusions

These results provide additional evidence of the harmful effects of sugar cane burning on respiratory functioning, specifically a “dose” response based on the number of acres burned as an indicator of volume of smoke. Maui is a small island with a complex environment that includes vog and other potential triggers of respiratory distress. New technologies may offer better detection of chemical markers of pollution; however, this study demonstrated that modern technology of satellite mapping can help control for confounders. While additional research will advance scientific understanding of the relationship between air pollution and health, public health officials should consider taking action based on the accumulation of research to date.
